# *Camellia sinensis* Aqueous Extract: A Promising Candidate for Hepatic Eimeriosis Treatment in Rabbits

**DOI:** 10.3390/ph16111598

**Published:** 2023-11-13

**Authors:** Hanadi B. A. Baghdadi, Mohamed Abdo Rizk

**Affiliations:** 1 Biology Department, College of Science, Imam Abdurrahman Bin Faisal University, Dammam 31441, Saudi Arabia; hbbaghdadi@iau.edu.sa; 2 Basic and Applied Scientific Research Center, Dammam 31441, Saudi Arabia; 3 Department of Internal Medicine, Infectious and Fish Diseases, Faculty of Veterinary Medicine, Mansoura University, Mansoura 35516, Egypt

**Keywords:** *Eimeria stiedae*, rabbit, green tea leaves, treatment, biochemical parameters, liquid chromatography–mass spectrometry, energy-dispersive X-ray spectroscopy

## Abstract

*Eimeria stiedae* (*E. stiedae*) is a common coccidian species that infects the liver and causes economic losses for the rabbit industry. This study aimed to determine the efficiency of green tea aqueous extract (GTE) as a natural treatment for eimeriosis caused by *E. stiedae*. Male rabbits *Cuniculus L*. (Oryctolagus) of the New Zealand White rabbit strain (4–4.5 months) were used, as they are suitable for research and conducting experiments. Thirty rabbits were allocated into six groups, with five rabbits in each group; the G1 group (non-infected untreated) served as a negative control group; the G2 group was not infected and treated with 250 mg GTE; the G3 group was not infected and treated with 500 mg GTE; the G4 group was untreated and was infected with 3 × 10^4^ Sporulated *E. stiedae* oocysts, which served as a positive control group; the G5 group was infected and treated with 250 mg GTE; and the G6 group was infected and treated with 500 mg GTE. The hematological and biochemical analyses of each group of rabbit sera were carried out. Phytochemical analysis was performed to evaluate the active components in GTE leaves using the following methods: IR spectroscopy, liquid chromatography–mass spectrometry (LC-MS), energy-dispersive X-ray spectroscopy (EDX), and scanning electron microscopy. The infected rabbit groups treated with GTE at both doses of 250 and 500 mg/kg exhibited a significant decrease in the extent of *E. stiedae* oocyst shedding compared with the infected untreated group at 14, 21, and 28 days post-infection. Also, treatment with green tea showed improvement in liver weight compared with the enlarged livers of infected, untreated rabbits. The disturbance in serum liver enzymes’ gamma-glutamyl transferase (GGT) and aspartate aminotransferase (AST/GOT) levels, as well as serum glucose, potassium, uric acid, cholesterol, and urea levels, were improved after the treatment of infected rabbit groups with green tea compared with the infected untreated group. Moreover, in this study, the images of the egg stages of the parasite were taken using a fluorescence microscope at 25 µm and 26 µm magnifications. This study provides promising results for the effective cell absorption of the aqueous extract of green tea, which was confirmed in the analyzed images using a scanning electron microscope at 5 µm and 20 µm magnifications.

## 1. Introduction

Rabbit farming has tremendous potential in developing countries to improve food safety and quality. Because of short pregnancy and pronounced prolificacy, rabbits are extremely productive. Rabbit meat is highly nutritious, juicy, and cholesterol-free, and it is high in calcium, vitamins, and minerals. Rabbits raised in backyards provide smallholder farmers with additional income and increase the food of provincial and urban individuals [1]. Today, rabbits are systematically reared on a vast scale, with global rabbit meat production reaching 1.8 million metric tons a year. Rabbit meat production is, in decreasing order, concentrated in Asia (48.8%), Europe (28.4%), the Americas (18.1%), and Africa (4.7%) [1]. China is the major rabbit meat producer (735,021 metric tons per year), mainly for export, followed by Italy, Spain, Egypt, and France (262,436; 67,775; 56,338; and 52,955 metric tons per year, respectively) [1]. In Italy, rabbit farming is the fourth-leading zootechnical sector, accounting for 9% of the gross domestic product [2]. Animal diseases are one of the general factors that pose restrictions on widespread rabbit farming [1]. Protozoan parasites are subject to these restrictions. In this regard, coccidiosis is a highly contagious, widespread enteric protozoal disease of rabbits that is caused by *Eimeria stiedae (E. stiedae)*. It is a major problem that causes significant financial losses on rabbit farms [3]. *Eimeria stiedae* infection was recorded, either clinically or microscopically, in rabbits from different countries, such as Taiwan, Iraq, the Kingdom of Saudi Arabia, Iran, India, Australia, Portugal, Poland, Brazil, Kenya, and Egypt, using parasitological or histopathological techniques [4]. *Eimeria* species are extremely host-, organ-, and tissue-specific; they are the cause of hepatic coccidiosis and have been associated with increased mortality [5]. Young kittens between the first and fourth months after birth are more susceptible to the disease than older rabbits, who seem to be more immune to infections. The disease spreads to the unaffected animal once it consumes contaminated material such as contaminated feed, water, and bedding [6]. Rabbits acquire the disease after eating sporulated oocysts. The disease’s general clinical signs include anorexia, dullness, reduced food intake, rough hair coat, brown diarrhea or constipation, emaciation, ascites, icterus, a distended abdomen, and ultimately death within 3–4 days [7]. Natural infections of *E. stiedae* can induce liver enlargement; the size and weight of the livers of dead animals increase due to the intense proliferation of biliary epithelia, resulting in the pressure atrophy of the hepatic epithelial cell and the obstruction of the bile ducts, and this may lead to the death of the infected rabbits [8]. The diagnosis of infection is primarily based on clinical signs and the detection of *E. stiedae* oocysts in feces [9]. Alterations in hematological and biochemical profiles, lipid peroxidation analysis, ultrasonographic and pathological images, and molecular techniques such as PCR are commonly used to diagnose the infection [10]. Unfortunately, hepatic coccidiosis is difficult to treat and may not be eliminated. Only the first 5–6 days of the anticoccidiosis medication are effective for rabbits even though mortality and diarrhea might still be present in the subsequent days [4]. Right now, the commonly used drugs for this infection in the field are diclazuril, toltrazuril, or toltrazuril in combination with ivermectin [11,12]. The currently used chemical drugs are expensive, have side effects, and do not fully treat the infection. Therefore, in recent years, attention has been drawn to the use of low-cost alternatives that can be easily obtained and that have no side effects on the animal or the environment. In this regard, herbal therapy may be an alternative. Following this line of investigation, in the present study, we evaluated the anticoccidial efficacy of green tea (*Camellia sinensis*) aqueous extract in *E. stiedae*-infected rabbits. 

In recent times, there has been considerable interest in green tea (*Camellia sinensis*) in both scientific and consumer circles due to its numerous potential health advantages, including its potential to address problems like metastasis in lung and breast cancer, as well as its positive impact on reducing blood cholesterol levels [13,14]. Previous studies have demonstrated the antileishmanial [15] and antiacanthamoeba [16] activities of green tea. However, the anticoccidial effect of green tea has not yet been evaluated. 

## 2. Results

### 2.1. Green Tea Aqueous Extract’s Active Components

The element content of green tea was determined using EDX analysis, and the results are expressed in atomic percentage. Although MN is a heavy metal in green tea, its content is 10.470%, while macroelements K, Ca, and P are 52.682%, 19.946%, and 4.075%, respectively. Trace elements of Fe (2.581%), Zn (0.318%), and Cu (0.425%) were also detected.

**Figure 1:**
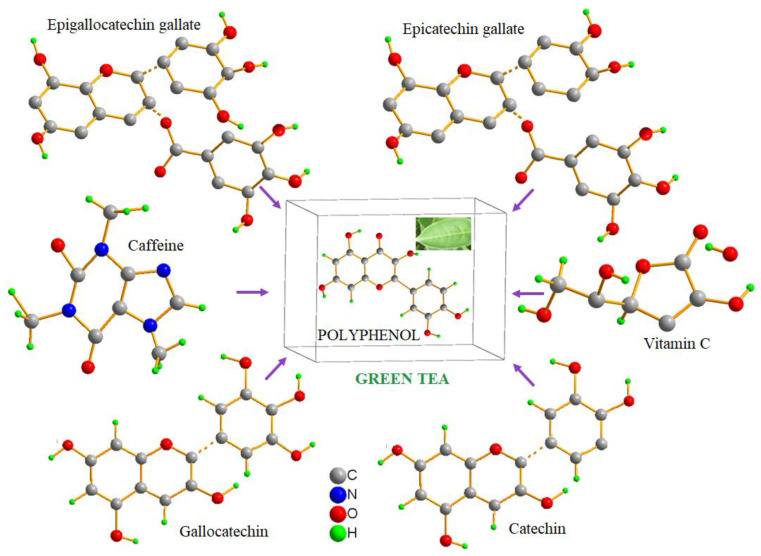
The molecular configurations of the compounds in the structure of green tea. Green tea aqueous extract is rich in polyphenolic catechisms, mainly epicatechin gallate and epigallocatechin gallate. Epicatechin gallate, which constitutes between 50 and 80 percent of all the catechins in green tea leaves, is the most prevalent catechin.

The analysis of the green tea extract using liquid chromatography–mass spectrometry (LC-MS) revealed the presence of multiple compounds, primarily polyphenolic catechisms, the main contributor to the antimicrobial activity of green tea. The most abundant among the catechisms found in the green tea extract was epicatechin gallate (ECG) with the chemical formula C_22_H_18_O_10_ and *m*/*w* of 442, followed by epigallocatechin gallate (EGCG) with the chemical formula C_22_H_18_O_11_ and *m*/*w* of 459 (Figure 1 and Figure S1). FTIR absorbance was also measured using a Fourier transform infrared spectrophotometer showing the characteristic bands (Figure S2).

### 2.2. XRD Analysis of the Green Tea Extract

In this analysis, we used a Shimadzu X-ray diffractometer, 7000, Q30545200239 (SHIMADZU, Tokyo, Japan). The XRD data of green tea samples were obtained from a Shimadzu powder diffractometer with Ni filter CuKα λ = 1.5406 Å radiation and a scan speed of 0.2°/min in the 10°, £ 2θ£ 80° range at room temperature. The crystalline phases were identified by comparing the diffraction patterns with those of the standard powder XRD files (JCPDS (Joint Committee on Powder Diffraction Standards) and COD (Crystallographic Open Database)).

**Figure 2:**
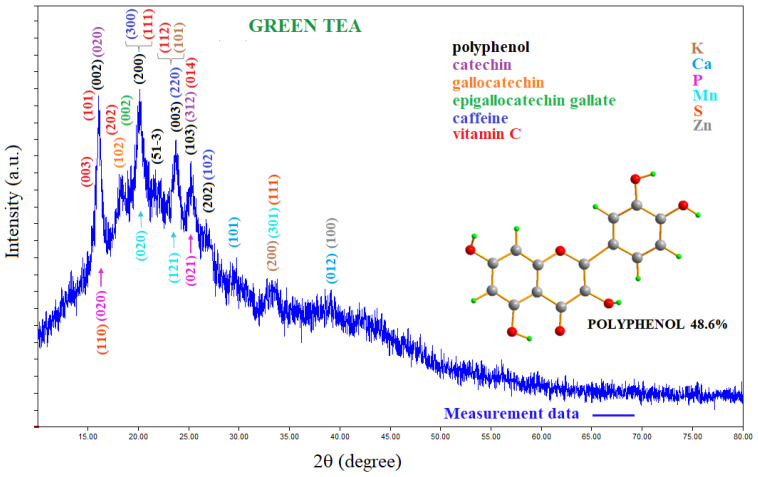
XRD pattern of green tea. EDX analysis results indicate that green tea aqueous extract is a rich source of K, Ca, P, Fe, Zn, and Cu.

From the analysis of the X-ray diffraction pattern, it was found that green tea contains polyphenol (COD: 96-721-1989, COD: 96-221-9197, COD: 96-402-9823, and COD: 96-221-2711) [17,18,19,20]; catechin (C) (COD: 96-410-1989) [21]; epigallocatechin gallate (EGCG) (COD: 96-155-1602) [22]; epicatechin gallate (ECG) (COD: 96-402-9823) [19]; and gallocatechin (GC) (COD: 96-722-3915) [23] groups. Figure S1 shows these compounds in green tea as molecular configurations. Based on the X-ray diffraction pattern, it also contains some minerals such as caffeine (COD: 96-705-0548, COD: 96-210-0203, COD: 96-705-0551, and COD: 96-221-9224) [24,25,26]; vitamin C (COD: 96-230-0647) [27] and vitamin K (COD: 96-900-8541) [28]; Ca (COD: 96-901-2731) [28]; P (COD: 96-151-2530) [29]; Mn (COD: 96-151-2521) [30]; Zn (COD: 96-151-2554) [31]; and S (COD: 96-901-4402) [32]. Figure 2 shows the X-ray diffraction pattern of green tea.

As can be seen from Figure 2, for the polyphenol group, 15.918°, 20.370°, 23.343°, 23.974°, 25.145°, and 26.858° Bragg diffraction angles (002), (200), (51-3), (003), (103), and (202) correspond to the hkl Miller indices, respectively. The (020) hkl Miller indices correspond to the 16,158° Bragg angle for catechin, and (002) hkl Miller indices correspond to the 15,918° Bragg angle for epigallocatechin gallate. It is seen from Figure S2 that the diffraction peaks of polyphenol, catechin, epigallocatechin gallate, epicatechin gallate, and gallocatechin groups overlap with each other. All of these groups are subgroups belonging to the same family, and the overlapping of diffraction patterns is expected. The results of the analysis reveal that green tea contains polyphenols at a percentage of 48.6%. This is in line with other studies reporting that 40% of green tea contains polyphenols [33,34]. In addition, as determined from Figure S2 and the data cards given above, green tea was also found to contain caffeine; vitamins C and K; and Ca, P, Mn, Zn, and S minerals (Figure 2).

### 2.3. Green Tea Aqueous Extract Inhibited the Multiplication of E. stiedae

Comparing the number of *E. stiedae* oocysts shed by infected untreated and infected green tea-treated rabbit groups at 14, 21, and 28 days post-infection (PI) revealed a marked decrease in the number of *E. stiedae* oocysts shed per gram of feces, from about 177,500 in the infected untreated group to 161,000 and 132,900 in the infected groups treated with green tea at doses of 250 and 500 mg/kg, respectively, after 14 days PI. In comparison, at 21 days PI, oocyst shedding decreased from 132,900.00 in the infected untreated group to 25,500 and 20,400 in the infected groups treated with green tea at doses of 250 and 500 mg/kg, respectively. At 28 days PI, oocyst shedding decreased from 500,000 in the infected untreated group to 3000 and 1200 in the infected groups treated with green tea at doses of 250 and 500 mg/kg, respectively. The ANOVA test revealed that the *p* value was 0.075 (Table 1).

### 2.4. Green Tea Aqueous Extract Improved the Liver Weight of the Infected Rabbit

After the end of the experiments (28 days PI), all rabbits were sacrificed, and the livers from all investigated groups were weighed. The infected untreated (G4) rabbits exhibited a higher increase in liver weight (138 g) than normal liver weight from non-infected (G1) rabbits (5.6 g). Treatment with green tea at both doses of 250 and 500 mg/kg resulted in a decrease in liver weight to 70 and 62 g, respectively, compared with the enlarged livers (138 g) obtained from the infected untreated group (G4). The ANOVA test revealed that the *p* value was significant (=0.95) (Table 2).

### 2.5. Green Tea Aqueous Extract Significantly Improved the Selected Biochemical Variables in Rabbits Infected with E. stiedae

There were obvious alterations in levels of serum liver enzymes, gamma-glutamyl transferase (GGT), glutamate oxaloacetate transaminase (GOT), and glutamate pyruvate transaminase (GPT) enzymes, and in addition, there was also disturbance in serum glucose, potassium (K), uric acid, cholesterol, and urea levels in the infected rabbit groups (G4) compared with the normal non-infected control group (G1). The treatment with green tea at both doses of 250 and 500 mg/kg resulted in a substantial improvement in all levels of these biochemical parameters, which almost reached the normal levels in the non-infected groups (Table 3). When comparing the infected and treated groups, the infected group revealed a significant (*p*) value of 0.95 (Table 3).

## 3. Discussion

One of the most significant diseases affecting the rabbit production industry is rabbit hepatic coccidiosis. In the present study, the prevalence of *Eimeria stiedae* infection among domestic rabbits in the Eastern Province was determined, and it was 34.88%, which is nearly similar to that determined in Saudi Arabia (32.24%) [35].

Notably, rabbits treated with green tea exhibited a normal postmortem and microscopical architecture of the liver, indicating its protective effect against *E. stiedae* infection. Additionally, macroscopic findings of the liver collected from treated rabbits confirmed the protective effect of green tea. This protective efficacy might be attributed to the inhibitory effect of green tea extract on lipid peroxidation, which could decrease the strength of the inflammatory response [36].

In the current study, in vivo anticoccidial effects of green tea extract were measured in terms of different parameters such as *E. stiedae* oocysts per gram of feces (OPG), a significant decrease was observed in the number of oocytes shed per gram of feces in the infected treated group. Comparable results were obtained by Abu El Ezz et al. [37,38,39] in rabbits experimentally infected with *E. stiedae*.

Notably, the rabbits treated with green tea at both doses of 250 and 500 mg/kg exhibited a significant improvement in the levels of serum glucose (GLU), potassium (K), uric acid, cholesterol, and urea, almost reaching the normal levels in the non-infected group. The results of the EDX analysis performed in the present study indicate that green tea aqueous extract is a rich source of biologically active compounds, such as macroelements K, Ca, and P, and trace elements of Fe, Zn, and Cu, which have antioxidant and antimicrobial activities. Also, the LC-MS analysis of the green tea aqueous extract revealed the presence of multiple compounds, mainly the polyphenolic catechisms epicatechin gallate (ECG) and epigallocatechin gallate (EGCG). The most abundant catechin type found in green tea is epicatechin gallate (ECG), which constitutes about 50–80% of the total catechins in the leaves. In a previous study, Winiarska-Mieczan et al. [40] found high contents of these aforementioned substances, with antioxidative, anti-inflammatory, and immunomodulatory properties. Subsequently, the observed improvement in the body weights, macroscopical findings, microscopical liver architecture, and estimated biochemical variables of the treated rabbits might be attributed to the richness of the used extract with these substances. In this regard, green tea is actually a functional food because of its high polyphenol content (namely EGCG, quercetin, theaflavin, thearubigin, and tannic acid), which are compounds with potent antioxidant qualities. Phenolic substances have antioxidative characteristics because they can (1) remove reactive oxygen species (ROS); (2) reduce ROS formation by chelating trace elements and limiting the activity of oxidative enzymes; and (3) boost the activity of endogenous antioxidants [40]. As EGCG has up to eight -OH groups in its chemical composition, it has a particularly potent antioxidative effect [40]. In addition to probably acting via mechanisms that either directly or indirectly control the development of enzymatic antioxidants, catechins primarily function by transporting H+ ions [41]. Due to its capacity to donate an electron or hydrogen atom, quercetin has antioxidative capabilities that enable it to neutralize singlet oxygen (1O2), O2•–, OH•, LOO•, NO, and ONOO– [40]. Thus, by blocking the activity of enzymes involved in ROS generation (such as oxidases) and enzymes that use NADPH as a cofactor, quercetin is able to neutralize ROS [40].

Notably, the anti-inflammatory properties of polyphenols may also contribute to the therapeutic benefits of green tea leaves. The main impact of polyphenols on the progression of inflammation involves their capacity to impede the production of proinflammatory cytokines, such as INF-γ, TNF-α, and chemokines, in diverse cell types [42]. Moreover, polyphenols exhibit anti-inflammatory properties on multiple fronts, primarily by blocking NF-κB and controlling mitogen-activated protein kinase (MAPK), iNOS, arachidonic acid, cyclooxygenase-2 (COX-2), and lipoxygenase (LOX), as well as by reducing reactive oxygen species (ROS) synthesis in comparison to reactive nitrogen species [40].

The limitations of this study should be noted. Although this study investigates the potential efficacy of green tea leaf aqueous extract in the treatment of hepatic eimeriosis, further studies are required to confirm liver pathology using histological examination and to determine the effect of the used herbal extract on other biochemical variables such as bilirubin. Also, further study is needed to determine the optimal dosage and treatment duration of green tea aqueous extract. Additionally, more in-depth studies are required to determine the potential mechanisms through which GTE may exert its anti-Eimeria effects.

## 4. Materials and Methods

### 4.1. Animals

At the animal house of Theodor Bilharz Research Institute in Egypt, thirty male New Zealand White rabbits, aged 6 weeks and weighing about 1.3 ± 0.07 kg, were raised in metal battery cages with a metallic grid on the bottom, keeping rabbits from coming into contact with their feces, under appropriate and stable healthy environmental conditions with a temperature of 22–25 °C, humidity 55–60%, ventilation, and 12 h of light and 12 h of darkness [43]. The rabbits were fed commercial pellet feed containing all the nutrients that were obtained from the Shuwayer, and clean drinking water was provided to the animals. The animals were left for ten days before the start of the experiment to acclimate to the place and the surrounding conditions [44].

The absence of *E. stiedae* and other coccidian oocysts preceding the trial was affirmed via fecal examination. All animals were weighed and numbered before the start of the experiment to determine the appropriate dose for each animal. Then, the weight of the rabbits was recorded every week during the experimental stages.

### 4.2. Laboratory Examination

Individual rabbit stool samples were taken from the rectum and placed in tiny (2″ × 2″) plastic containers. The samples were subsequently processed in the lab for the detection and identification of coccidian oocysts using direct smear and saturated sugar floatation techniques [45]. Using the McMaster technique, coccidial oocysts per gram (OPG) of feces were counted to gauge the severity of infection before and after therapy. Oocysts were sporulated at 25 °C in a 2.5% potassium dichromate solution [46]. *Eimeria *stiedae** oocysts were detected after examination with a light microscope, and the size of the unsporulated and sporulated was measured using a calibrated ocular micrometer.

### 4.3. Experimental Infection of Rabbits with Isolated and Identified E. stiedae

The *Eimeria stiedae* strain was obtained from a local field and isolated from naturally infected rabbits with irregular yellowish-white nodules scattered on the liver surface. Sample collection, concentration, and purification, as well as the sporulation of oocysts from gallbladders and necrotic hepatic lesions, were carried out according to the flotation method as previously described [47]. Briefly, the livers and gallbladders were removed, minced, and digested in 0.25% trypsin in normal saline. Then, the digested materials were sieved and washed several times via centrifugation at 2000 rpm for 10 min each. The oocysts were allowed to sporulate via incubation for 3 days in a 2.5% potassium dichromate solution at 30 °C. Then, the sporulated oocysts were counted and kept at 4 °C until use in experimental infection.

In this experiment, after 7 days of adaptation, the thirty laboratory rabbits were divided into six groups, with five rabbits in each group, as follows: The G1 group (non-infected, untreated) served as the negative control group; the G2 group was non-infected and treated with 250 mg of green tea; the G3 group was non-infected and treated with 500 mg of green tea; the G4 group was untreated and was infected with sporulated *E. stiedae* oocysts, which served as the positive control group; the G5 group was infected and treated with 250 mg of green tea; and the G6 group was infected and treated with 500 mg of green tea (Supplementary Table S1).

Sporulated oocysts were washed four times with distilled water and dialyzed using tap water for 24 h. The inoculum was concentrated to 15,000 sporulated oocysts per 1 mL. Each rabbit from the 3 experimental infected groups (G4, G5, and G6) was orally infected with 2 mL of 3 × 10^4^ sporulated E. stiedae oocysts via a stomach tube. Rabbits from the non-infected group (G1) were treated with saline in the same way [48]. The oocysts and sporulated oocyst stages were photographed using a Bio-Rad ZOE Fluorescent Cell Imager at different magnifications, as shown in Figure S3.

### 4.4. Herbal Extract (Green Tea^®^)

Green tea leaves (*Camellia sinensis*) obtained from a local market were used in two doses of 250 and 500 mg/kg of body weight in the form of an aqueous suspension dissolved in 5 mL of distilled water. The green tea leaf powder was dissolved in 50 mL of distilled water and incubated for 3 days at 30 °C. The resulting product was then filtered using Whatman filter paper No. 1. Using a rotary evaporator (BUICHI^®^RotavaporR-200/205, Flawil, Switzerland) under decreased pressure at 40 °C and a freeze-dry vacuum system (Labconco, Kansas City, MO, USA), the resulting extract was concentrated and lyophilized [49]. Then, 100 mg of crude extract was dissolved in 1 mL of DMSO to obtain a stock aqueous extract of green tea leaf solution.

The active components of green tea leaves were evaluated via phytochemical analysis with the following methods: IR spectroscopy, liquid chromatography–mass spectrometry (LC-MS), energy-dispersive X-ray spectroscopy (EDX), and scanning electron microscopy [23]. In this study, green tea extract particles were photographed using a scanning electron microscope to determine the shape and size of these particles and the extent of their permeability and effectiveness in treatment, as shown in Figure S4.

### 4.5. In Vivo Treatment

The therapeutic nontoxic doses from the aqueous extract of green tea leaves were administered to the treated animals after the 14th day of infection, which was the day on which the coccidian oocysts appeared in the stool. Animals had a recovery period of two weeks after the end of the treatment period to ensure the effectiveness of the treatment.

### 4.6. Hematological and Biochemical Analyses

Blood samples were collected from either infected or non-infected rabbits for the assessment of hematological and biochemical parameters. Using a 2 mL sterile disposable syringe, blood was obtained from ear veins. The blood was put into a plain vacutainer tube, allowed to stand in the refrigerator for 15 min, and centrifuged at 3500× *g* for 5 min for serum collection.

Serum was used to detect the activities of liver enzymes, mainly gamma-glutamyl transferase (GGT), glutamate oxaloacetate transaminase (GOT), and glutamate pyruvate transaminase (GPT) enzymes, and in addition, serum glucose (GLU), potassium (K), uric acid (UA), cholesterol (CHOL), and urea (Ur) were measured using colorimetric methods [21] according to the instructions of the kit manufacturer (Bio-Systems/Barcelona, Spain).

### 4.7. Statistical Analysis

Data analyses were performed using a statistical software program (JMP for Windows Version 5.1; SAS Institute, Cary, NC, USA). The mean values and standard deviation for each assessed variable were calculated. For the evaluation of treatment results, repeated-measure MANOVA over treatment and time was used to determine the main effect of the drug and time. Wilks’ Lambda test was also performed to evaluate within-group interactions and obtain evidence of time–group interactions. While Wilks’ Lambda test indicated a statistically significant difference between groups, one-way ANOVA and Tukey–Kramer HSD post hoc multiple-comparison tests were used to identify which group was statistically different from the rest. Differences between means at *p* < 0.05 were considered significant.

## 5. Conclusions

As rabbits are an important food source, especially for the elderly, due to the low cholesterol level in their meat, it is important to search for appropriate methods of treatment of *Eimeria stiedae* infection in rabbits. So, in light of the current study, it was found that the infection of *Eimeria stiedae* in the Eastern Province’s pet animals affects the productivity of this type of animal and reflects negatively on the economic sources of food in the region. The findings of the current investigation suggest that changes in clinical, hematological, and biochemical parameters can facilitate the prediction of how the infection caused by *E. stiedae* will progress. The results of the present study provide evidence of the potential of GTE as a natural and effective treatment option for hepatic eimeriosis in rabbits. Also, the study recommends using green tea as an effective method of the treatment and prevention of infection from this type of parasite, with the need for additional studies to determine the exact ingredients and dosages appropriate for the age and weight of each animal.

## Figures and Tables

**Table 1:** Number of oocyst/gram in feces in infected and infected treated groups.

Days Post-Infection	Infected Untreated Group (G4)	Infected Treated Group (250 mg/kg) (G5)	Infected Treated Group (500 mg/kg) (G6)
**14**	177,500.0 ± 6379.21	161,000.00 ± 56,262.28	132,900.00 ± 13,472.97 *
**21**	132,900.00 ± 13,472.97	25,500.0 ± 6678.73 *	20,400.0 ± 7713.19 *
**28**	500,000.00 ± 19,720	3000.00 ± 152.75 *	1200.00 ± 152.05 *

Each value represents (mean ± SD). Asterisks indicate a significant difference (* *p* < 0.05) between the treated and untreated groups; *p* values for time and Wilks’ Lambda for treatment–time interaction for all treated groups were <0.05.

**Table 2:** Liver weight of infected and infected treated rabbit groups.

Days Post-Infection	Non-Infected Group (G1)	Infected Untreated (G4)	Infected Treated (250 mg/kg) (G5)	Infected Treated (500 mg/kg) (G6)
**14**	3.66 ± 0.577	78.00 ± 2.64	79.00 ± 1.00	78.33 ± 1.52
**21**	4.00 ± 0.00	108.33 ± 24.66	68.33 ± 7.63 *	66.66 ± 7.63 *
**28**	3.66 ± 0.577	159.00 ± 52.84	60.00 ± 13.22 *	61.66 ± 16.07 *

Each value represents (mean ± SD). Asterisks indicate a significant difference (* *p* < 0.05) between the treated and the untreated groups; *p* values for time and Wilks’ Lambda for treatment–time interaction for all treated groups were <0.05.

**Table 3:** Hematological and biochemical variables (U/L) estimated in different groups.

Enzymes and Biochemical Parameters	Non-Infected Group (G1)	Infected Untreated (G4)	Infected Treated (250 mg/kg) (G5)	Infected Treated (500 mg/kg) (G6)
Glucose	9.31 ± 0.24	7.04 ± 0.25	9.19 ± 0.25	9.34 ± 0.22
Gamma-glutamyl transferase (GGT)	4.68 ± 0.14	4.46 ± 0.15	4.94 ± 0.08	4.79 ± 0.14
Glutamate oxaloacetate transaminase (GOT)	4.98 ± 0.41	44.59 ± 5.54	22.73 ± 3.14 *	21.21 ± 1.48 *
Glutamate pyruvate transaminase (GPT)	4.95 ± 0.09	46.38 ± 5.00	40.70 ± 2.15	20.07 ± 1.49 *
Potassium (K)	5.67 ± 0.36	6.544 ± 0.633	4.93 ± 0.08	5.00 ± 0.14
Uric acid	2.81 ± 0.09	4.27 ± 0.34	3.19 ± 0.13	4.100 ± 0.10
Cholesterol	96.10 ± 1.32	104.10 ± 3.57	99.00 ± 0.57	97.93 ± 0.90
UREA	40.93 ± 4.12	63.94 ± 4.16	49.60 ± 1.11 *	44.98 ± 1.18 *

Each value represents (mean ± SD). Asterisks indicate a significant difference (* *p* < 0.05) between the treated and the untreated groups.

## Data Availability

Data is contained within the article and supplementary material.

## Supplementary Materials

The following supporting information can be downloaded at: https://www.mdpi.com/article/10.3390/ph16111598/s1, Figure S1: Liquid Chromatography Mass Spectrometry (LC-MS) analysis of green tea. Figure S2: FT-IR of Green Tea extract using a Fourier Transform infrared spectrophotometer. Figure S3: Fluorescence microscopic image of Oocyst and sporulated oocyst of Eimeria stiedae Bio-Rad ZOE Fluorescent Cell Imager is used. Figure S4: Scanning Electron Microscopic image of green tea extraction. Table S1: Rabbits grouping for the experiment design.
