# Supplementary material for: Camellia sinensis Aqueous Extract: A Promising Candidate for Hepatic Eimeriosis Treatment in Rabbits
Source: Pharmaceuticals (Basel). 2023 Nov 13;16(11):1598. doi: 10.3390/ph16111598 (PMC10674767; doi:10.3390/ph16111598)
Supplement: Supplementary file 1 [file pharmaceuticals-16-01598-s001.zip › pharmaceuticals-2664421-supplementary.pdf]

Supplementary data

Title

A *Camellia sinensis* aqueous extract: a promising therapeutic candidate on hepatic Eimeriosis in Rabbits

Figure S1. Liquid Chromatography Mass Spectrometry (LC-MS) analysis of green tea.

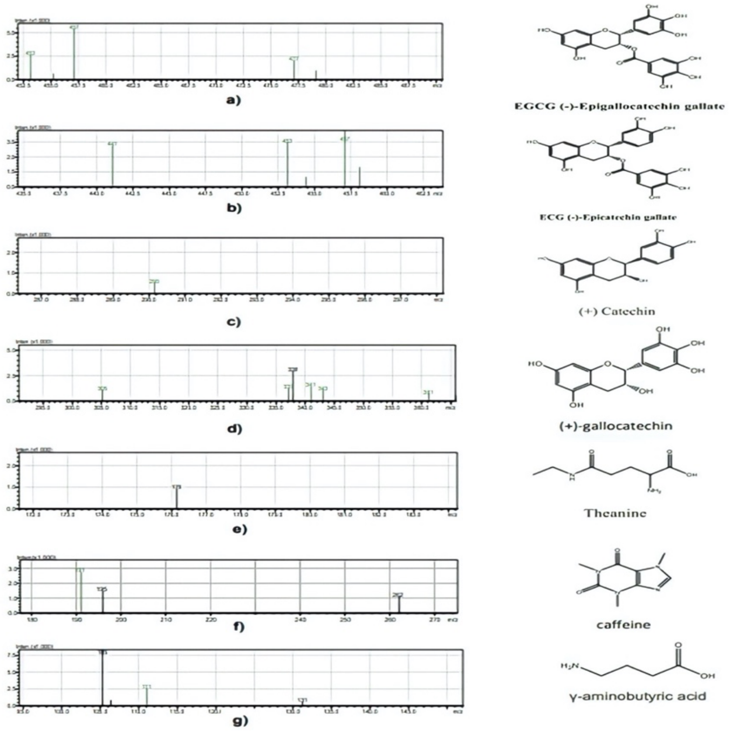

Figure S2. FT-IR of Green Tea extract using a Fourier Transform infrared spectrophotometer.FTIR absorbance was measured using a Fourier Transform infrared spectrophotometer (IR-SPIRIL-T), A22415801447AE, Shimadzu with a Smart ATR sampling accessory the FTIR scanning was performed from 4000 cm-1 to 400 cm1 the number of scans was 24. The characteristics bands by FT-IR are as follows; In green tea IR spectrum, at 3340 cm–1 band stretching vibration of O–H groups and N-H for water, alcohol phenols, and amines. The C–H stretch at 2920 and O-H stretch at 2858 cm–1 for alkanes and carboxylic acid respectively. The band at 1729 cm–1 C=O Stretch of aromatic ring & band at 1638 cm–1 in polyphenols. For the amide in protein, the C-N stretch gives the band at 1371 cm–1. The C–O–C stretching in polysaccharides gives a band at 1729 cm–1 and C–O stretching in amino acids causes a band at

1020 cm–1. band at 832 cm–1 of C–H plane bending. the IR spectrum of green tea observed that rich in polysaccharides, polyphenols, carboxylic acids, amino acids, and proteins.

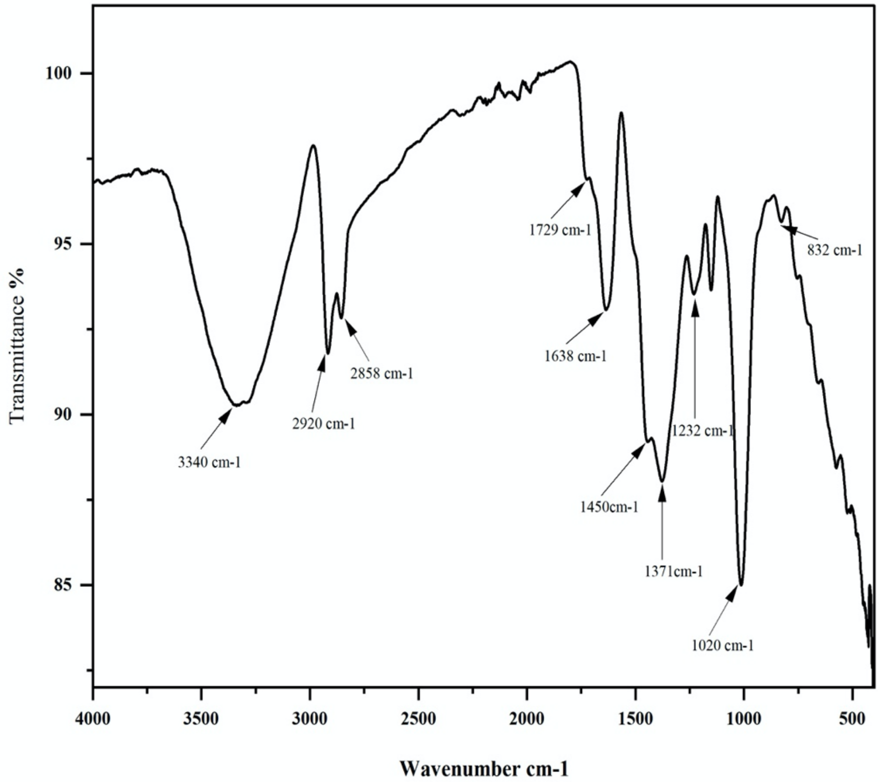

Figure S3. Fluorescence microscopic image of Oocyst and sporulated oocyst of *Eimeria stiedae.* Bio-Rad ZOE Fluorescent Cell Imager is used.

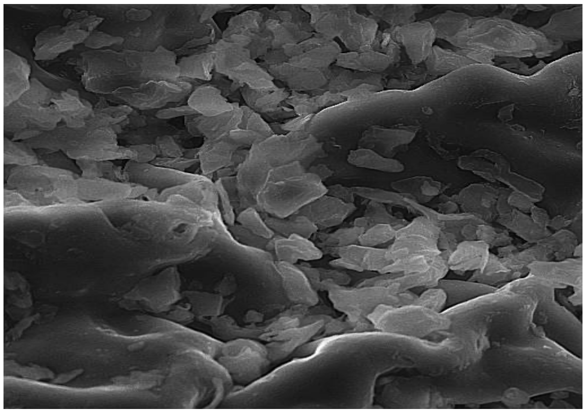

Figure S4. Scanning Electron Microscopic image of green tea extraction.

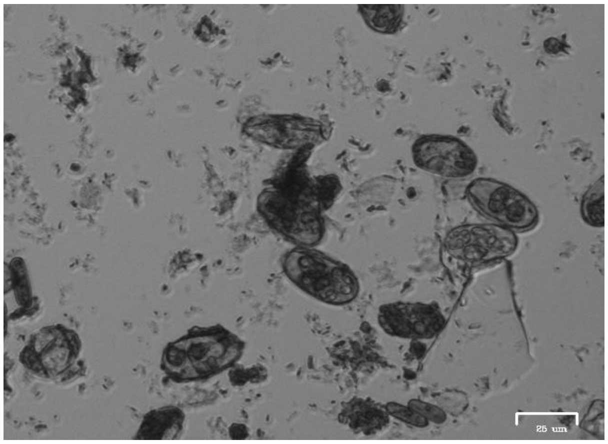

Supplementary Table S1 . Rabbits grouping for the experiment design.

Rabbit groups No.	Group name
**G1**	Non-infected untreated (-ve control)
**G2**	Non-infected and treated with 250 mg GTE
**G3**	Non-infected and treated with 500 mg GTE
**G4**	Infected and untreated (+ve control)
**G5**	Infected and treated with 250 mg GTE
**G6**	Infected and treated with 500 mg GTE
